# Lipid profile, cardiovascular disease and mortality in a Mediterranean high-risk population: The ESCARVAL-RISK study

**DOI:** 10.1371/journal.pone.0186196

**Published:** 2017-10-18

**Authors:** Domingo Orozco-Beltran, Vicente F. Gil-Guillen, Josep Redon, Jose M. Martin-Moreno, Vicente Pallares-Carratala, Jorge Navarro-Perez, Francisco Valls-Roca, Carlos Sanchis-Domenech, Antonio Fernandez-Gimenez, Ana Perez-Navarro, Vicente Bertomeu-Martinez, Vicente Bertomeu-Gonzalez, Alberto Cordero, Manuel Pascual de la Torre, Jose L. Trillo, Concepcion Carratala-Munuera, Salvador Pita-Fernandez, Ruth Uso, Ramon Durazo-Arvizu, Richard Cooper, Gines Sanz, Jose M. Castellano, Juan F. Ascaso, Rafael Carmena, Maria Tellez-Plaza, on behalf of ESCARVAL Study Group

**Affiliations:** 1 Catedra de Medicina de Familia, Miguel Hernandez University, San Juan de Alicante, Spain; 2 Department of Internal Medicine, Hospital Clinico de Valencia, Valencia, Spain; 3 INCLIVA Research Institute, Valencia, Spain; 4 CIBERObn, ISCIII, Madrid, Spain; 5 Department of Preventive Medicine and Public Health, University of Valencia Medical School. Valencia, Spain; 6 Health Surveillance Department, Mutual Society of Castellon. Department of Medicine. Jaume I University. Castellon, Spain; 7 Department of Medicine, University of Valencia, Valencia, Spain; 8 Health Centre of Beniganim, Generalitat Valenciana, Beniganim, Valencia, Spain; 9 Health Centre of Algemesi, Generalitat Valenciana, Algemesi, Valencia, Spain; 10 ESCARVAL Project, Valencia, Spain; 11 Department of Cardiology, Hospital Universitario San Juan de Alicante, San Juan de Alicante, Spain; 12 Department of Clinical Medicine, Miguel Hernández University, San Juan de Alicante, Spain; 13 Biomedical Informatics. Electronic Health Record Office. Conselleria de Sanitat. Valencia, Spain; 14 Department of Pharmacy, Hospital Clinico de Valencia, Valencia, Spain; 15 Clinical Epidemiology and Biostatistics Unit, Complexo Hospitalario Universitario A Coruña (CHUAC), SERGAS, Universidad de A Coruña, A Coruña, Spain; 16 Pharmacy Management. Conselleria de Sanitat. Valencia, Spain; 17 Department of Public Health Sciences, Stritch School of Medicine, Loyola University Chicago, Maywood, IL, United States of America; 18 National Cardiovascular Research Center. Centro Nacional de Investigaciones Cardiovasculares (CNIC), Madrid, Spain; 19 HM Hospitales, Hospital Universitario HM Monteprincipe, Madrid, Spain; 20 Service of Endocrinology and Nutrition, Hospital Clínico de Valencia. University of Valencia, Valencia, Spain; 21 INCLIVA Research Institute. Ciber de Diabetes y Enfermedades Metabólicas (CIBERDEM), Carlos III. Valencia, Spain; 22 Institute for Biomedical Research. Hospital Clinic de Valencia, Valencia, Spain; 23 Department of Environmental Health Sciences, Johns Hopkins University Bloomberg School of Public Health, Baltimore, United States of America; University College London, UNITED KINGDOM

## Abstract

**Introduction:**

The potential impact of targeting different components of an adverse lipid profile in populations with multiple cardiovascular risk factors is not completely clear. This study aims to assess the association between different components of the standard lipid profile with all-cause mortality and hospitalization due to cardiovascular events in a high-risk population.

**Methods:**

This prospective registry included high risk adults over 30 years old free of cardiovascular disease (2008–2012). Diagnosis of hypertension, dyslipidemia or diabetes mellitus was inclusion criterion. Lipid biomarkers were evaluated. Primary endpoints were all-cause mortality and hospital admission due to coronary heart disease or stroke. We estimated adjusted rate ratios (aRR), absolute risk differences and population attributable risk associated with adverse lipid profiles.

**Results:**

51,462 subjects were included with a mean age of 62.6 years (47.6% men). During an average follow-up of 3.2 years, 919 deaths, 1666 hospitalizations for coronary heart disease and 1510 hospitalizations for stroke were recorded. The parameters that showed an increased rate for total mortality, coronary heart disease and stroke hospitalization were, respectively, low HDL-Cholesterol: aRR 1.25, 1.29 and 1.23; high Total/HDL-Cholesterol: aRR 1.22, 1.38 and 1.25; and high Triglycerides/HDL-Cholesterol: aRR 1.21, 1.30, 1.09. The parameters that showed highest population attributable risk (%) were, respectively, low HDL-Cholesterol: 7.70, 11.42, 8.40; high Total/HDL-Cholesterol: 6.55, 12.47, 8.73; and high Triglycerides/HDL-Cholesterol: 8.94, 15.09, 6.92.

**Conclusions:**

In a population with cardiovascular risk factors, HDL-cholesterol, Total/HDL-cholesterol and triglycerides/HDL-cholesterol ratios were associated with a higher population attributable risk for cardiovascular disease compared to other common biomarkers.

## Introduction

Between 1990 and 2013, age-standardized death rates from cardiovascular and circulatory diseases fell by 22% in Western Europe, while ischemic heart disease and stroke remain the main causes of years of life lost [1]. Therefore, primary prevention strategies based on identification of total cardiovascular risk are essential for cardiovascular disease (CVD) control. Risk assessment tools to estimate the patient's 10-year risk of developing CVD have become the cornerstone to identify high-risk people for primary prevention. Both the Framingham-based equations [2] and the European Systematic COronary Risk Evaluation (SCORE) algorithm [3] (the most widely used for clinical practice guidelines), include total cholesterol and high density lipoprotein cholesterol (HDL-C) as the main lipid parameters. The Framingham risk equations were developed during the peak incidence of CVD in the United States, and they perform well in similar populations but may overestimate risk by up to 50% in contemporary European populations, where the incidence of CVD is lower [4]. On the other hand, the SCORE risk prediction chart assesses risk in people up to 65 years of age, but estimation of absolute risk of coronary heart disease and CVD in the elderly is needed for targeted preventive activities, particularly in low-incidence, low-mortality Southern European countries, where life expectancy continues to increase [5].

Low density lipoprotein cholesterol (LDL-C) is the predominant cholesterol-carrying lipoprotein, and is considered to be the main atherogenic lipoprotein. However other lipoproteins such as (HDL-C or very low density lipoprotein have shown repeatedly to play a role in atherogenesis. Recent epidemiological data suggests that isolated low HDL-C in people with normal LDL-C and triglyceride (TG) levels is equivalent to elevated LDL-C as a coronary risk factor [6–8]. Moreover, low HDL-C levels and the ratio of total serum cholesterol (TC) to HDL-C levels have been introduced in the novel CVD risk scores, such as the QRISK and QRISK2 [9]; the latter being currently recommended by the National Clinical Guideline Centre for Cardiovascular Risk Assessment for primary prevention of CVD [10].

There is limited clinical data however, that prospectively evaluates the association of lipid markers and cardiovascular risk in high-risk populations. In addition, the potential impact on attributable risk of hypothetical interventions targeting novel lipid markers has not been fully explored in contemporary populations with additional cardiovascular risk factors such as hypertension or diabetes. The aim of the present study was to prospectively estimate and compare the attributable risk associated with several lipid markers for all-cause mortality and hospitalization due to CVD in participants with at least one of the three major cardiovascular risk factors: hypertension, diabetes or dyslipidemia, receiving usual care and participating in the ESCARVAL-RISK project [11] “EStudio CARdiometabolico VALenciano” in a Mediterranean population.

## Methods

ESCARVAL-RISK is an observational cohort study in individuals with cardiovascular risk factors (hypertension, dyslipidemia, or diabetes mellitus) and free of previous CVD. Treatment for hypertension, diabetes or dyslipidemia, as well as other concomitant diseases, was left to the discretion of primary care physicians and patients were treated according to current clinical guidelines. Therefore, the ESCARVAL-RISK study was specifically designed to investigate associations between three major cardiovascular risk factors and CVD in the real world setting of clinical practice.

### Study population

The Valencia Community is a Mediterranean region located on the east coast of Spain, with a total population of 4.980.689 according to the 2015 census. The cohort was recruited from a sample of patients receiving healthcare by the Valencia Health System. Every user of this system has a unique patient identifier, corresponding to a centralized, individual electronic clinical record. The unique patient identifier allows linkage between relevant clinical databases where various variables were collected. Detailed information about the sample size recruitment has been published elsewhere [11].

Briefly, we included 73,302 participants of both sexes, aged 30 years or older with a diagnosis of hypertension, diabetes mellitus, and/or dyslipidemia, with no previous cardiovascular events who attended a primary healthcare center for routine health services. Of the total population, there was missing data on body weight for 12,209 participants, on serum creatinine for 5,175 participants, and on other variables of interest for 4,456 participants. After excluding these participants, our final sample size included 51,462 participants. Information was collected from ABUCASIS, which is the electronic health record (EHR) that registers patient data in the Valencia region.

### Baseline data collection

Data on age, sex, smoking and medication for treating hypertension, diabetes, and hypercholesterolemia was collected from the EHR. Blood pressure was measured up to three times on the same day in the sitting position following the European guidelines on CVD prevention in clinical practice [12]. Hypertension was defined as a mean systolic blood pressure ≥140 mm Hg, a mean diastolic blood pressure ≥90 mm Hg, a recorded physician diagnosis, or medication use. Diabetes was defined as a non-fasting glucose level of ≥200 mg/dl, a recorded physician diagnosis, medication use, or an HbA1c ≥ 6.5%. TC was measured enzymatically using the Cholesterol High Performance reagent (Roche Diagnostics). HDL-C was measured using a direct HDL reagent (Roche Diagnostics). LDL-C was calculated using the Friedwald formula [13]. High cholesterol was defined as a serum total cholesterol >200 mg/dL, recorded diagnosis or medication use. Non-HDL cholesterol was measured according to the difference between TC and HDL-C. Triglycerides were measured using Hitachi 704 Analyzer which is serviced by Roche Diagnostics (formerly Boehringer-Mannheim Diagnostics), Indianapolis. Also non-HDL minus LDL-cholesterol was calculated. Body mass index (BMI) was calculated by dividing measured weight in kilograms by height in squared meters and obesity was defined as a BMI ≥30 kg/m^2^.

### Mortality and hospitalization follow-up

The follow up period was from January 2008 to December 2012. Participants were followed up until the first episode of hospitalization for CHD or stroke or for death. Data on all-cause mortality was collected. At the time of inclusion, information about cardiovascular risk factors and their active treatments as well as smoking habit and biochemistry lab values were collected from the EHR. Mortality data were obtained from death certificates registered in the Spanish National Death Index. The cause of hospitalization was determined by the codes assigned according to the International Classification of Diseases, 10^th^ Revision (ICD-10). Cause-specific hospitalization was defined as the first in-hospital admission for CHD (ICD codes 410–414) or stroke (ICD codes 430–438, 444). Cardiovascular hospitalizations or mortality during follow-up were assessed by annual mortality and morbidity surveillance reviews of hospitalization and death records. Follow-up data was available for 99.8% of subjects for mortality and for 99.2% of subjects for morbid events. Time to first event was calculated as the difference between the date of the baseline examination and the date of the hospital admission, date of death or 31 December 2012, whichever occurred first.

The study was conducted according to the standards of the International Guidelines for Ethical Review of Epidemiological Studies (Council for International Organizations of Medical Sciences-CIOMS-Geneva, 1991). The ESCARVAL-RISK study [11] was reviewed and approved by the Valencia Committee for Ethics and Clinical Trials of the Center for Public Health Research (DGSP-CSISP). Patient data collected from the ABUCASIS EHR during the study were anonymized, making it impossible to use the information to identify the patients. The data generated during the study were handled according the Spanish Law 5/1999 and corresponding regulations. All of the researchers with access to study data were required to sign a document guaranteeing confidentiality. No informed consent from patients was required.

### Statistical analysis

Age-adjusted rates for mortality and cardiovascular hospitalization end-points were estimated using Poisson regression for individual data with over-dispersion correction. Multi-adjusted rate differences were estimated from semi-parametric Aalen additive hazard models. Statistical models were adjusted for age (continuous-modelled as restricted cubic splines with five knots), sex (male, female), BMI (continuous), hypertension (no, yes), hypertension medication (no, yes), diabetes (no, yes), diabetes medication (no, yes), smoking status (never, former, current), high LDL-C (<130 mg/dL, ≥130 mg/dL), low HDL-C (≤40 mg/dL for men; ≤50 mg/dL for women) and use of cholesterol-lowering medication (no, yes). Adjusted population attributable risks (PARs) for dichotomous lipid biomarkers were calculated by using the standard formula PAR = 1 – Σ_*j*_Σ_*i*_ p_*ij*_ / RR_*i| j*_ [14]. In this formula, the subscript *i* denotes one of two categories of the lipid biomarkers (with each participant classified according to the presence of the corresponding biomarker being used to calculate the PAR), the subscript *j* is an index for all strata obtained after cross-classifying the study sample for all adjusted covariates, p_*ij*_ is the proportion of total cases in the study population in each stratum after cross-classifying the dichotomous biomarker category and all adjusted covariates, and RR_*i|j*_ is the adjusted hazard ratio for the endpoint of interest comparing participants with and without the biomarker in stratum *j* of covariates, from Cox proportional hazards regression. Adjusted PARs represent the estimated fraction of deaths that would be avoided in the population, had participants above a given cut-off of the biomarker been below it, assuming that the effects are causal and that other risk factors remain unchanged. We created 55,000 bootstrap samples to obtain the standard errors and 95% confidence intervals for PAR.

## Results

### Participant characteristics

A total of 51,462 patients with at least one cardiovascular risk factor were included in the study. The main characteristics of the study population, grouped by the study endpoints, are shown in Table 1. Hypertension was present in 79% and diabetes in 37% of the participants. Thirty percent were receiving lipid-lowering drug treatment. During an average follow-up of 3.2 years, the EHR recorded 919 deaths (80,705.3 person-years at risk) 1666 hospitalizations for CHD (78,643.85 person-years at risk) and 1510 stroke hospitalizations (79,130.76 person-years at risk). Age-adjusted rates (deaths/10,000 person-years) of CVD and mortality endpoints by CVD risk factors per quartiles of each lipid parameter are described in Table 2.

**Table 1:** Participant characteristics according to presence or absence of all-cause mortality and CVD hospitalization end-points.

		All-cause mortality		CHD hospitalization		Stroke hospitalization	
	Overall (N = 51,462)	No (N = 50,543)	Yes (N = 919)	No (N = 49,796)	Yes (N = 1666)	No (N = 49,952)	Yes (N = 1510)
Age, mean years (SD)	62.65 (12.07)	62.45 (12.02)	73.37 (10.21)	62.5 (12.1)	67.19 (10.36)	62.41 (12.05)	70.44 (9.89)
Men, %	47.59	47.26	65.61	47.07	63.09	47.35	55.56
BMI, kg/m^2^, mean (SD)	29.53 (4.83)	29.54 (4.83)	29.19 (4.89)	29.52 (4.84)	29.87 (4.59)	29.54 (4.84)	29.29 (4.55)
Obesity, %	42.09	42.12	40.48	41.99	44.90	42.15	40.07
Former smoking, %	20.78	20.66	27.53	20.39	32.59	20.67	24.57
Current smoking, %	22.22	22.26	20.46	22.39	17.29	22.35	17.95
Diabetes, %	37.20	36.88	54.41	36.62	54.44	36.76	51.59
Glucose lowering medication, %	19.39	19.51	12.95	19.00	31.15	19.14	27.95
Systolic blood pressure, mean mmHg (SD)	136.24 (18.19)	136.17 (18.16)	140.19 (19.64)	136.11 (18.14)	140.32 (19.26)	136.11 (18.14)	140.75 (19.17)
Diastolic blood pressure, mean mmHg (SD)	79.36 (10.85)	79.41 (10.84)	76.71 (10.83)	79.4 (10.83)	78.4 (11.37)	79.39 (10.83)	78.52 (11.41)
Hypertension, %	78.98	78.78	90.42	78.55	92.14	78.59	92.05
Antihypertensive medication, %	43.56	43.95	22.20	43.10	57.20	43.27	53.18
Chronic kidney disease, %	15.01	14.69	32.43	14.69	24.31	14.66	26.36
Total cholesterol, mean mg/dL (SD)	211.01 (40.97)	211.26 (40.93)	197.58 (41.02)	211.35 (40.84)	201.02 (43.61)	211.25 (40.9)	203.12 (42.49)
HDL-cholesterol, mean mg/dL (SD)	52.7 (13.94)	52.74 (13.93)	50.55 (13.98)	52.83 (13.94)	48.86 (13.2)	52.76 (13.95)	50.8 (13.38)
Non-HDL-cholesterol, mean mg/dL (SD)	158.31 (39.41)	158.52 (39.39)	147.04 (38.7)	158.52 (39.3)	152.16 (42)	158.49 (39.36)	152.32 (40.49)
LDL-cholesterol, mean mg/dL (SD)	126.2 (34.33)	126.36 (34.31)	117.62 (34.37)	126.4 (34.25)	120.06 (36.16)	126.34 (34.3)	121.47 (35.03)
Triglycerides, mean mg/dL (SD)	149.69 (100.01)	149.89 (100.29)	138.46 (82.37)	149.47 (100.13)	156.38 (96.15)	149.86 (100.51)	143.95 (81.65)
Ratio total/HDL-cholesterol	4.22 (1.2)	4.22 (1.2)	4.12 (1.17)	4.22 (1.2)	4.34 (1.27)	4.22 (1.2)	4.2 (1.19)
Ratio triglycerides/HDL-cholesterol	3.26 (3.01)	3.26 (3.02)	3.11 (2.69)	3.25 (3.01)	3.63 (3.07)	3.26 (3.03)	3.18 (2.36)
Dyslipidemia, %	88.23	88.39	79.22	88.04	93.76	88.20	89.01
Lipid lowering medication, %	30.28	30.60	12.73	29.80	44.60	30.09	36.76

CVD: cardiovascular disease; CHD: coronary heart disease; SD: statistical deviation; BMI: body mass index; HDL: High density lipoprotein; LDL: low density lipoprotein

**Table 2:** Age and sex-adjusted rates of all-cause mortality and CVD hospitalization by quartile of serum lipids.

	Quartile				p-value
	Q1	Q2	Q3	Q4	
**Total cholesterol**					
Median (range), mg/dL	165 (118, 183)	165 (184, 210)	223 (211, 237)	257 (238, 329)	
All-cause mortality					
Cases (person-years)	341 (43,630.74)	255 (43,449.59)	184 (41,295.09)	139 (41,238.03)	
Rate	60.3	55.5	51.0	49.5	0.027
CHD hospitalization					
Cases (person-years)	609 (42,385.83)	429 (42,671.84)	306 (40,716.51)	322 (40,637.52)	
Rate	126.0	97.7	80.6	95.2	<0.001
Stroke hospitalization					
Cases (person-years)	504 (42,683.04)	388 (42,774.50)	321 (40,668.79)	297 (40,714.40)	
Rate	98.3	85.0	84.7	93.9	0.361
**HDL cholesterol**					
Median (range), mg/dL	38 (27, 43)	38 (44, 51)	56 (52, 61)	69 (62, 98)	
All-cause mortality					
Cases (person-years)	315 (45,630.38)	228 (42,053.36)	191 (42,115.12)	185 (39,814.59)	
Rate	66.0	52.9	46.0	50.7	<0.001
CHD hospitalization					
Cases (person-years)	633 (44,381.46)	435 (41,240.43)	337 (41,467.21)	261 (39,322.61)	
Rate	135.8	103.5	83.7	73.6	<0.001
Stroke hospitalization					
Cases (person-years)	487 (44,749.58)	378 (41,346.15)	364 (41,439.38)	281 (39,305.62)	
Rate	110.8	90.4	86.3	71.9	<0.001
**Non-HDL cholesterol**					
Median (range), mg/dL	115 (74, 131)	115 (132, 156)	169 (157, 183)	204 (184, 276)	
All-cause mortality					
Cases (person-years)	325 (43,768.99)	253 (42,218.93)	192 (42,588.25)	149 (41,037.27)	
Rate	58.0	55.9	50.0	52.7	0.154
CHD hospitalization					
Cases (person-years)	558 (42,614.69)	420 (41,425.83)	352 (41,964.77)	336 (40,406.42)	
Rate	117.6	98.0	88.0	96.4	<0.001
Stroke hospitalization					
Cases (person-years)	477 (42,867.75)	383 (41,571.73)	339 (41,923.08)	311 (40,478.17)	
Rate	92.1	85.2	85.2	99.8	0.492
**LDL cholesterol**					
Median (range), mg/dL	88 (50, 102)	88 (103, 124)	135.75 (125, 148)	166 (149, 224)	
All-cause mortality					
Cases (person-years)	314 (41,816.10)	246 (41,215.15)	203 (43,304.18)	156 (43,278.03)	
Rate	60.9	56.0	50.4	49.7	0.015
CHD hospitalization					
Cases (person-years)	577 (40,679.63)	397 (40,445.12)	342 (42,666.73)	350 (42,620.22)	
Rate	128.4	95.6	83.1	94.4	<0.001
Stroke hospitalization					
Cases (person-years)	460 (40,965.45)	369 (40,527.55)	370 (42,657.15)	311 (42,690.59)	
Rate	95.5	85.2	90.2	91.1	0.606
**Non-HDL minus LDL cholesterol**					
Median (range), mg/dL	16.6 (7.6, 21.0)	16.6 (22.0, 30.0)	35 (30.6, 40.3)	48.25 (41.0, 93.0)	
All-cause mortality					
Cases (person-years)	308 (51,216.52)	239 (42,451.48)	206 (38,554.97)	166 (37,390.47)	
Rate	52.6	52.7	53.8	58.5	0.319
CHD hospitalization					
Cases (person-years)	449 (50,328.06)	431 (41,582.03)	391 (37,851.46)	395 (36,650.16)	
Rate	85.5	99.8	102.5	118.1	<0.001
Stroke hospitalization					
Cases (person-years)	448 (50,354.07)	420 (41,656.99)	335 (37,963.04)	307 (36,866.61)	
Rate	81.4	94.8	87.7	101.0	0.013
**Triglycerides**					
Median (range), mg/dL	74 (43, 91)	74 (92, 124)	147 (126, 176)	232 (179, 628)	
All-cause mortality					
Cases (person-years)	256 (43,230.56)	249 (43,280.85)	222 (42,904.17)	192 (40,197.86)	
Rate	52.8	53.2	52.0	59.2	0.347
CHD hospitalization					
Cases (person-years)	354 (42,575.48)	400 (42,462.60)	426 (42,111.74)	486 (39,261.89)	
Rate	80.9	91.6	99.6	130.7	<0.001
Stroke hospitalization					
Cases (person-years)	355 (42,606.01)	400 (42,519.69)	410 (42,135.18)	345 (39,579.85)	
Rate	77.5	88.2	96.0	101.2	<0.001
**Ratio total/HDL cholesterol**					
Median (range), mg/dL	2.96 (2.11, 3.35)	2.96 (3.38, 4.03)	4.42 (4.06, 4.86)	5.58 (4.92, 8.29)	
All-cause mortality					
Cases (person-years)	261 (42,536.33)	220 (42,720.99)	238 (42,608.40)	200 (41,747.72)	
Rate	53.8	49.1	55.9	58.0	0.276
CHD hospitalization					
Cases (person-years)	370 (41,798.70)	392 (41,961.01)	435 (41,806.98)	469 (40,845.02)	
Rate	87.2	91.8	102.1	119.8	<0.001
Stroke hospitalization					
Cases (person-years)	386 (41,761.15)	366 (42,108.46)	388 (41,901.78)	370 (41,069.34)	
Rate	81.3	81.3	92.4	107.3	<0.001
**Ratio TG/HDL cholesterol**					
Median (range), mg/dL	1.20 (0.58, 1.57)	1.20 (1.61, 2.44)	3.05 (2.49, 3.84)	5.49 (3.95, 17.88)	
All-cause mortality					
Cases (person-years)	229 (42,638.68)	232 (43,056.65)	241 (42,876.82)	217 (41,041.30)	
Rate	51.5	49.4	54.6	61.6	0.041
CHD hospitalization					
Cases (person-years)	286 (42,105.98)	395 (42,282.16)	466 (42,014.74)	519 (40,008.84)	
Rate	70.3	91.1	107.7	133.2	<0.001
Stroke hospitalization					
Cases (person-years)	321 (42,083.52)	409 (42,283.75)	398 (42,119.24)	382 (40,354.22)	
Rate	73.2	90.2	92.0	107.3	<0.001

CVD: cardiovascular disease; CHD: coronary heart disease; HDL: High density lipoprotein; LDL: low density lipoprotein

Age and sex-adjusted rates: events/10,000 person-years

### Lipid parameters, mortality and hospitalization for CHD or stroke rates

**Fig 1:**
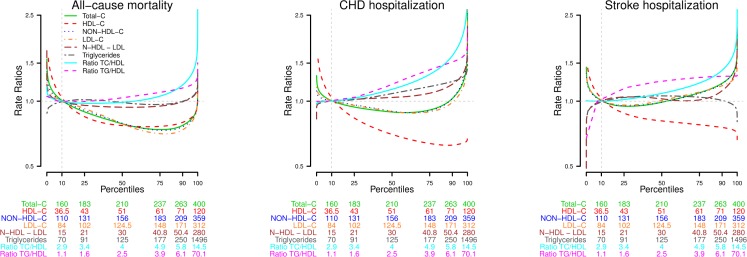
Age and sex-adjusted rates ratios for all-cause mortality and CVD hospitalization by serum lipid levels. Models are adjusted for age, sex, smoking status (never, former, current), obesity (no, yes), diabetes (no, yes), hypertension (no, yes), chronic kidney disease (no, yes), anti-hypertensive medication (no, yes), glucose lowering medication (no, yes), lipid-lowering medication (no, yes). Models for specific lipid biomarkers have been additionally adjusted as follows: 1) Total-cholesterol is further adjusted by HDL ≤ 40 for men and ≤ 50 for women (no, yes); 2) HDL-cholesterol is further adjusted by LDL.C ≥ 130 mg/dL (no, yes); 3) Non-HDL-cholesterol is further adjusted by HDL ≤ 40 for men and ≤ 50 for women (no, yes); 4) LDL-cholesterol is further adjusted by HDL ≤ 40 for men and ≤ 50 for women (no, yes); 5) Non-HDL minus LDL-cholesterol is further adjusted by HDL ≤ 40 for men and ≤ 50 for women (no, yes) and LDL-C ≥ 130 mg/dL (no, yes); 6) Triglycerides is further adjusted by total cholesterol > 200 mg/dL (no, yes) and HDL ≤ 40 for men and ≤ 50 for women (no, yes); 7) Total cholesterol/HDL is further adjusted by total cholesterol (mg/dL); and 7) Triglycerides/HDL is further adjusted by total cholesterol (mg/dL).

Age-adjusted mortality rates (deaths/10,000 person-years) showed a significant positive association with the TG/HDL-C ratio and an inverse association with TC, HDL-C and LDL-C. Regarding the risk of hospitalization for CHD, a significant positive association was observed in non-HDL-C minus LDL-C, TG, TC/HDL-C ratio and TG/HDL-C ratio, and an inverse relationship with TC, HDL-C and LDL-C. For hospitalization due to stroke, there was a significant positive association with LDL-C, non-HDL-C minus LDL-C, TG, TC/HDL-C ratio and TG/HDL-C ratio, and an inverse association with HDL-C. After further adjustment for other cardiovascular risk factors, however, the association of elevated TC and LDL-C levels and CHD hospitalization was no longer significant, either in the relative or additive scales (Tables 3 and 4). For stroke, however, the association with LDL-C remained significant in fully adjusted models. The rate ratio and rate differences (95% CI) for all–cause mortality and CVD hospitalization after a 3.2-year follow-up comparing the 75^th^ versus 25^th^ percentile of lipid biomarkers concentrations are shown in the S1 Table, with consistent findings. Fig 1 shows the fully adjusted dose-response association between lipid values and risk of mortality and hospitalization due to CHD and stroke. A sensitivity analysis was performed in participants according to current lipid-lowering treatment (S1 Fig), but no relevant differences were found between groups.

**Table 3:** Rate differences after a 5-year follow-up by altered lipid levels.

	All-cause mortality		CHD hospitalization		Stroke hospitalization	
	Model 1	Model 2	Model 1	Model 2	Model 1	Model 2
**High total cholesterol** ^**a**^	-10.70 (-18.08, -3.33)	-16.36 (-24.15, -8.57)	-30.62 (-41.12, -20.12)	-5.76 (-16.65, 5.13)	-2.84 (-12.50, 6.82)	11.16 (0.95, 21.38)
**Low HDL-cholesterol**^**b**^	11.55 (4.10, 19.00)	7.79 (0.12, 15.46)	40.46 (30.20, 50.73)	26.55 (16.07, 37.03)	25.61 (15.45, 35.76)	17.96 (7.45, 28.47)
**High non-HDL-cholesterol**^**c**^	-18.16 (-37.71, 1.39)	-23.66 (-43.31, -4.02)	-47.98 (-75.79, -20.16)	-27.67 (-55.59, 0.25)	-26.68 (-51.56, -1.80)	-16.05 (-41.12, 9.03)
**High LDL-cholesterol**^**d**^	-8.32 (-15.04, -1.60)	-15.26 (-22.30, -8.22)	-22.51 (-31.72, -13.29)	-1.88 (-11.32, 7.57)	-3.56 (-12.62, 5.50)	6.91 (-2.46, 16.27)
**High non-HDL minus LDL-cholesterol**^**e**^	5.47 (-1.48, 12.41)	1.53 (-5.57, 8.63)	15.46 (5.53, 25.38)	5.58 (-4.55, 15.71)	3.80 (-5.38, 12.98)	-2.43 (-11.85, 6.99)
**High triglycerides**^**f**^	0.57 (-6.56, 7.70)	-2.02 (-9.69, 5.64)	25.44 (14.97, 35.91)	7.94 (-3.50, 19.37)	10.59 (1.08, 20.09)	-3.61 (-13.74, 6.53)
**High total/HDL-cholesterol**^**g**^	2.17 (-4.97, 9.30)	2.22 (-5.54, 9.99)	18.10 (7.37, 28.83)	23.14 (11.61, 34.67)	16.04 (6.24, 25.84)	14.55 (3.80, 25.31)
**High triglycerides/HDL-cholesterol**^**h**^	5.14 (-1.93, 12.21)	3.77 (-3.45, 10.98)	35.76 (25.64, 45.87)	22.13 (11.75, 32.51)	12.98 (3.90, 22.06)	4.25 (-5.29, 13.78)

CVD: cardiovascular disease; CI: confidence interval HDL: High density lipoprotein; LDL: low density lipoprotein

Rate Differences: events/10.000 person-years (95%CI)

Model 1 is adjusted for age and sex. Model 2 is Model 1 further adjusted for smoking status (never, former, current), obesity (no, yes), diabetes (no, yes), hypertension (no, yes), chronic kidney disease (no, yes), anti-hypertensive medication (no, yes), glucose-lowering medication (no, yes), lipid-lowering medication (no, yes). Models for specific lipid biomarkers have been additionally adjusted as follows

Total-cholesterol^**a**^ is further adjusted by HDL ≤ 40 for men and ≤ 50 for women (no, yes)

HDL-cholesterol^**b**^ is further adjusted by LDL ≥ 130 mg/dL (no, yes)

Non-HDL-cholesterol^**c**^ is further adjusted by HDL ≤ 40 for men and ≤ 50 for women (no, yes)

LDL-cholesterol^**d**^ is further adjusted by HDL ≤ 40 for men and ≤ 50 for women (no, yes)

Non-HDL minus LDL-cholesterol^**e**^ is further adjusted by HDL ≤ 40 for men and ≤ 50 for women (no, yes) and LDL-C ≥ 130 mg/dL (no, yes)

Triglycerides^**f**^ further adjusted by total cholesterol > 200 mg/dL (no, yes) and HDL ≤ 40 for men and ≤ 50 for women (no, yes)

Total cholesterol/HDL^**g**^ is further adjusted by total cholesterol (mg/dL); and

Triglycerides/HDL^**h**^ is further adjusted by total cholesterol (mg/dL).

**Table 4:** Population attributable risk (95% CI) for all-cause mortality and CVD hospitalization by altered lipid levels.

	Prevalence	All-cause mortality	CHD hospitalization	Stroke hospitalization
**High total cholesterol**^**a**^	60.19%			
RR		0.83 (0.73, 0.96)	0.95 (0.86, 1.06)	1.14 (1.02, 1.27)
PAR		-9.18 (-16.44, -2.12)	-2.32 (-7.41, 2.69)	6.60 (1.19, 11.91)
**Low HDL-cholesterol**^**b**^	31.61%			
RR		1.19 (1.04, 1.37)	1.31 (1.18, 1.45)	1.23 (1.1, 1.37)
PAR		5.32 (1.02, 9.64)	8.92 (5.41, 12.44)	6.64 (3.09, 10.20)
**High non-HDL-cholesterol**^**c**^	94.51%			
RR		0.84 (0.68, 1.05)	0.86 (0.72, 1.02)	0.9 (0.75, 1.09)
PAR		-16.71 (-41.12, 5.88)	-15.25 (-34.09, 2.50)	-9.85 (-29.16, 8.46)
**High LDL-cholesterol**^**d**^	43.99%			
RR		0.85 (0.74, 0.98)	0.98 (0.88, 1.09)	1.09 (0.98, 1.21)
PAR		-5.93 (-11.12, -0.70)	-0.76 (-4.54, 3.01)	3.14 (-0.94, 7.20)
**High Non-HDL minus LDL-cholesterol**^**e**^	51.14%			
RR		1.03 (0.9, 1.17)	1.08 (0.98, 1.19)	0.99 (0.89, 1.1)
PAR		1.08 (-4.68, 6.71)	3.58 (-1.18, 8.28)	-0.54 (-5.19, 4.15)
**High Triglycerides**^**f**^	36.12%			
RR		1.05 (0.91, 1.23)	1.11 (0.99, 1.24)	0.99 (0.88, 1.11)
PAR		1.65 (-3.18, 6.33)	3.95 (-0.26, 8.09)	-0.49 (-4.63, 3.62)
**High Total/HDL-cholesterol**^**g**^	29.46%			
RR		1.1 (0.94, 1.3)	1.31 (1.17, 1.47)	1.2 (1.06, 1.35)
PAR		2.20 (-1.49, 5.88)	7.10 (4.05, 10.17)	4.58 (1.44, 7.70)
**High triglycerides/HDL-cholesterol**^**h**^	39.42%			
RR		1.16 (1.01, 1.33)	1.28 (1.16, 1.42)	1.08 (0.97, 1.2)
PAR		4.96 (0.28, 9.61)	9.91 (5.85, 13.91)	2.81 (-1.27, 6.84)

CI: confidence interval; CHD: coronary heart disease; CVD: cardiovascular disease; PAR: population attributable risk; RR: rate ratio; HDL: High density lipoprotein; LDL: low density lipoprotein

Model 1 is adjusted for age and sex. Model 2 is Model 1 further adjusted for smoking status (never, former, current), obesity (no, yes), diabetes (no, yes), hypertension (no, yes), chronic kidney disease (no, yes), anti-hypertensive medication (no, yes), glucose lowering medication (no, yes), lipid-lowering medication (no, yes). Models for specific lipid biomarkers have been additionally adjusted as follows

Total-cholesterol^**a**^ is further adjusted by HDL ≤ 40 for men and ≤ 50 for women (no, yes)

HDL-cholesterol^**b**^ is further adjusted by LDL-C ≥ 130 mg/dL (no, yes)

Non-HDL-cholesterol^**c**^ is further adjusted by HDL ≤ 40 for men and ≤ 50 for women (no, yes)

LDL-cholesterol^**d**^ is further adjusted by HDL ≤ 40 for men and ≤ 50 for women (no, yes)

Non-HDL minus LDL-cholesterol^**e**^ is further adjusted by HDL ≤ 40 for men and ≤ 50 for women (no, yes) and LDL-C ≥ 130 mg/dL (no, yes)

Triglycerides^**f**^ is further adjusted by total cholesterol > 200 mg/dL (no, yes) and HDL ≤ 40 for men and ≤ 50 for women (no, yes)

Total cholesterol/HDL^**g**^ is further adjusted by total cholesterol (mg/dL); and

Triglycerides/HDL^**h**^ is further adjusted by total cholesterol (mg/dL).

### Disease burden associated with lipid parameters

Multi-adjusted differences in rate of events/10,000 person-years of mortality and CVD endpoints (attributable risk) are shown in Table 3. Low HDL-C and a high TC/HDL-C ratio were associated with the absolute risk of hospitalization for CHD and stroke. High TG/HDL-C also increased the absolute risk of hospitalization for CHD. The population attributable risks (PAR) associated with the lipid parameters are shown in Table 4. Low HDL-C, high TC/HDL-C and high TG/HDL-C were associated with a PAR for mortality of 5.3%, 2.2% and 5.0%, respectively. For CHD hospitalization the PAR was 8.9%, 7.1% and 9.9%, respectively. For stroke hospitalization, the attributable risk was low HDL-C (6.6%) and high TC/HDL-C (4.6%).

## Discussion

In this Mediterranean population with at least one major cardiovascular risk factor, HDL-C levels and the ratios of TC/HDL-C and TG/HDL-C were associated with all-cause mortality and risk of hospitalization due to CHD and stroke, while LDL-C was associated with stroke but not with CHD. These data were confirmed in a sensitivity analysis carried out in participants not receiving lipid-lowering therapy at baseline. The PARs associated with HDL-C, TC/HDL-C and TG/HDL-C ranged from 4.5% (CI95% 4.4–7.7; PAR of stroke associated to elevated TC/HDL-C) to 9.9% (CI95% 5.8–13.9; PAR of CHD associated with elevated TG/HDL-C).

### Characteristics of the study population and the source of data

Participants included in the analysis had a sociodemographic profile comparable to the overall selected population. The age and sex-adjusted rates in our population were lower compared to the United States and other European countries [4, 5], reflecting a country-specific profile of low cardiovascular risk, as supported by the SCORE study [3].

### Relationship between lipid parameters and cardiovascular risk in previous studies in Spain

The age-adjusted absolute rates for mortality or hospitalization for CHD or stroke by lipid values and indexes presented in this paper are consistent with previously published studies carried out in Spain and elsewhere. The relationship between lipid parameters and rates of cardiovascular risk has been explored previously in Spain, although these were performed in the 1990s. The ERICE [15] and the FRESCO [5] studies included data from 11 population cohorts in seven Spanish regions. The results of the FRESCO study were consistent with our finding that HDL-C was the lipid factor most strongly associated with cardiovascular risk. In the ERICE study, total cholesterol did not show a statistical association with cardiovascular events, however it was not sufficiently powered to assess the impact of HDL-C. Recent data from a study carried out in patients recently diagnosed with diabetes mellitus [16], identified the ratio of non-HDL-C to HDL-C as a significant predictor of cardiovascular events, while LDL-C and TC did not show significant associations.

### Lipid parameters as predictors of vascular events

Studies performed worldwide in different populations have found associations between cardiovascular events and low HDL-C [17–26]. Other studies have found associations with the TC/HDL-C ratio [27–29], yet others have showed conflicting data [30, 31].

It seems clear that low HDL-C is a strong and independent risk factor for CVD. HDL-C particles may act as a protective factor against atherosclerosis via multiple biological mechanisms [32]: effluxing cellular cholesterol, diminishing cellular death, decreasing vascular constriction, reducing inflammatory response, protecting from pathological oxidation, combating bacterial infection, lessening platelet activation, regulating gene expression by virtue of microRNAs, and improving glucose metabolism.

Data from the Jupiter Study [33] has shown that baseline LDL-C was not associated with CVD events. In another recent publication [34] of data from more than 350,000 people from three cohorts (REasons for Geographic And Racial Differences in Stroke [REGARDS], Kaiser Permanente Southern California [KPSC] and Atherosclerosis Risk In Communities [ARIC]) the results suggested that the association between LDL-C and CHD in contemporary studies may be diminished by the preferential use of statins in high risk individuals, while the association with HDL-related markers remains. While we have not found relevant differences between participants based on treatment at baseline (S1 Fig), we cannot rule out the presence of a time-varying residual confounding effect by statin use during the follow-up period in our study population.

It is also important to note that with regard to prediction of vascular events, the most commonly used predictive scales for cardiovascular risk (Framingham [2], SCORE [3]) consider total cholesterol, HDL cholesterol and the ratio of total cholesterol to HDL-C to be the strongest predictors. In the most recent and widely accepted QRISK2 [9], however, the lipid parameter included for cardiovascular risk calculation is the ratio TC/HDL-C. The NICE dyslipidemia guideline [10] recommends using the QRISK2 risk assessment tool to assess cardiovascular risk for the primary prevention of CVD in people aged 84 and younger. So ESCARVAL study results agree with these data in order to conclude that nowadays, lipid parameters as HDL-C or Total Cholesterol / HDL-C are more strongly associated with cardiovascular events than the most used in clinical practice as LDL-C, and are better predictors to estimate the cardiovascular risk, especially in high risk patients.

Prospective studies measuring not only HDL and LDL cholesterol levels, but also the number and size of particles, are needed to further elucidate the association between lipid particles and cardiovascular risk.

### Multi-adjusted rate differences in mortality or hospitalizations: PAR

In order to further explore the relationship between lipid parameters and cardiovascular risk, we analyzed the multi-adjusted rate difference in mortality and hospitalizations as well as the PAR by altered lipid levels (Table 3). This value can be interpreted as the average annual increase in mortality and risk of hospitalization due to a cardiovascular event on an absolute scale, attributable to the factor considered and the relative amount of avoidable deaths and hospitalization in the population studied, respectively.

The PAR provides an estimated measure of the public health impact of a potential intervention targeting specific risk factors, in the hypothetical scenario where the association of these markers and CVD risk is causal, and the other risk factors remain unchanged. For most of the evaluated endpoints (mortality and hospitalization for CHD or stroke), low HDL showed a higher PAR than the other lipid markers included in the study. Few studies have analyzed the impact of lipid particles and PAR, and their results are inconsistent. The Framingham Offspring study followed a cohort with a mean baseline age of 51 years for two decades, finding that low levels of HDL-C, high LDL-C and high levels of TG in any combination, were associated with increased CVD risk. In fact, the highest PARs were for the groups including high LDL-C, especially in the presence of concomitantly low HDL-C and/or high TG [35]. Another study, in which the incidence was similar to that of the United States in the 1970s, found that low HDL-C and high TC were associated with a similar PAR to the one found in our study [36]. Discrepancies on the impact of LDL-C or TC on cardiovascular risk may be explained by our selection criteria, which included a higher risk profile and higher use of statins. PAR for cardiovascular risk associated with lipid parameters in other contemporary studies are, however, scarce.

### Strengths and limitations

There are some limitations to the current study that should be mentioned. Firstly, the lower CVD risk inherent to the Mediterranean population could limit the generalizability of our results. In addition, the results apply only to individuals with at least one cardiovascular risk factor and cannot be extrapolated to the general population.

The results of this observational study should be considered within the advantages and limitations of registry-based data [37]: the use of EHRs offers a timely alternative and these databases provide a low-cost means of accessing rich longitudinal data on large populations for epidemiologic research. Using EHR for data collection reflects real clinical practice, in contrast to data from clinical trials. Another potential advantage, as in the current study, is the large number of participants and events, which provided enough statistical power and a valuable framework in which to assess the attributable risk of mortality, CHD, and stroke to cardiovascular risk factors in the short term in a real life setting.

The mean follow-up was 3.2 years. Although this is a relatively short time period, we evaluated a high risk study population, which resulted in 919 deaths (80,705.3 person-years at risk), 1666 hospitalizations for CHD (78,643.85 person-years at risk), and 1510 hospitalizations for stroke. Most cardiovascular risk scales predict cardiovascular risk at 10 years, which has been proposed as a limitation for high-risk populations. It has become increasingly clear that from a public health perspective and in clinical practice the need for shorter-term scales in order to intensify interventions, avert clinical inertia and improve therapeutic adherence. The lack of association between LDL-C and cardiovascular events in the current study could be influenced by a shorter time scale used, raising the question of whether LDL-C is a good short-term predictor of cardiovascular events in high-risk patients. In a study with longer follow-up period, the association between LDL-C and events may be prove to be stronger.

## Conclusions

**Fig 2:**
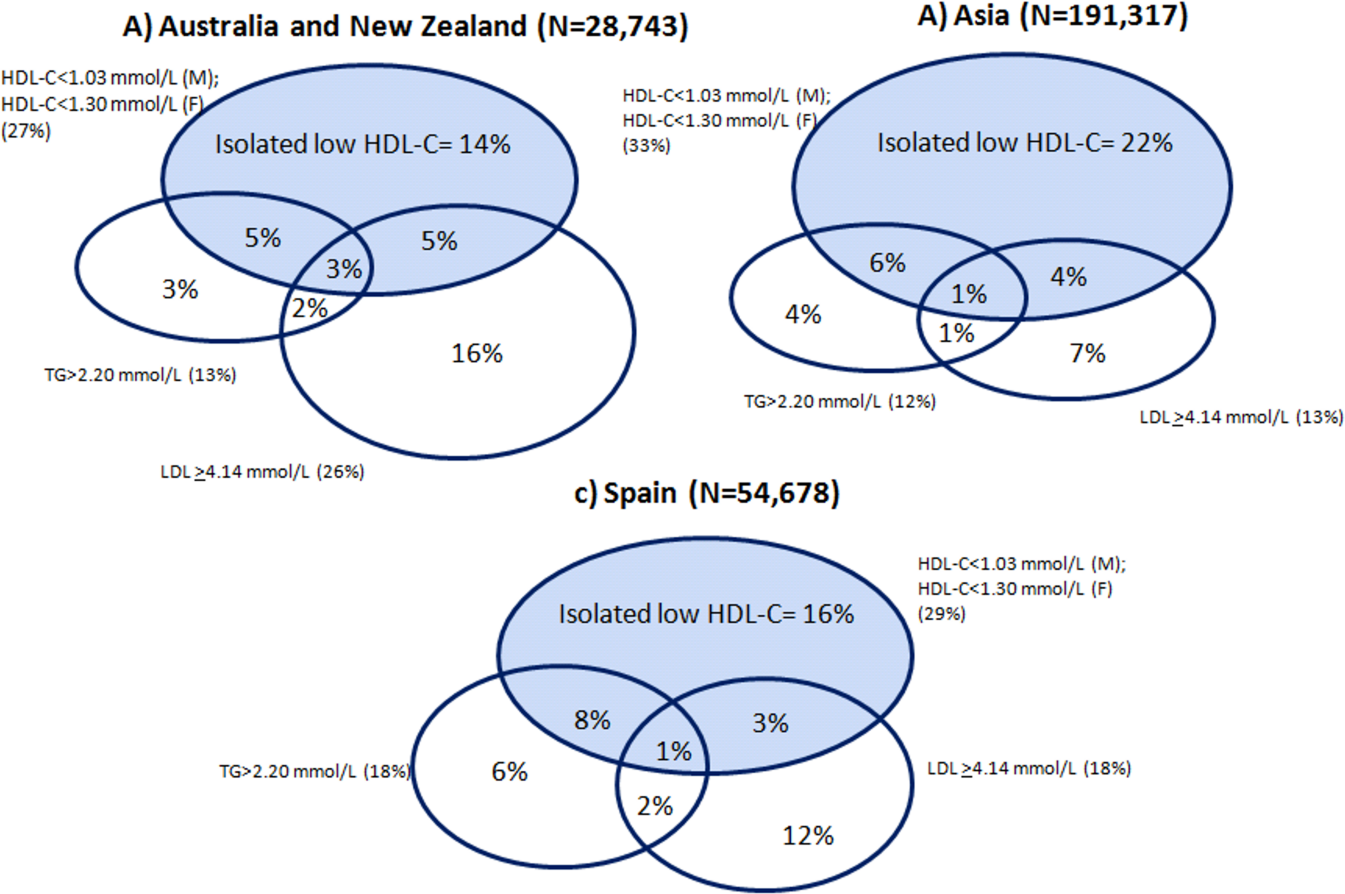
Proportion of patients with low HDL-cholesterol, including those with isolated low HDL-cholesterol. Comparison between Australian, Asian and Spanish cohorts. Completed from Huxley RR et al. Circulation. 2011;124:2056–2064) (M = Male; F = Female). Isolated low HDL-cholesterol: patients with normal levels of triglycerides and LDL-Cholesterol.

Clinical trials have clearly established that reduced LDL-C levels are associated with fewer cardiovascular events in both high- and low-risk populations. However, despite advances in research for prevention and acute treatment, including new therapeutic agents, cardiovascular disease is still the first cause of death in developed and developing countries. Identification of high risk patients is critical in order to propose effective prevention strategies. There is a significant proportion of patients with LDL-C levels within the normal range, whether in lipid-lowering therapy or not, in whom cardiovascular events do not appear to have been satisfactorily prevented [38]. Many individuals who reach LDL-C targets still possess an atherogenic lipid profile with residual risk. In a recent meta-analysis, around 15% of people from population cohorts in Asia had isolated low HDL-C (patients with normal levels of triglycerides and LDL-C) [39]. We found a similar proportion in our study (Fig 2). In light of these results, one should consider including low HDL, with normal TG and normal LDL as a higher risk category. Individuals exhibiting this form of lipid abnormality are at increased risk of CHD, but at the same time, are not likely to receive lipid-lowering medication according to current clinical guidelines, based on their levels of triglycerides and LDL-C. So, probably we are not identifying high risk patients properly. Nowadays, other lipid parameters in addition to LDL-C should be included for a more accurate evaluation of CV risk and to determine which patients must receive preventive treatment.

For patients with hypertension, diabetes or dyslipidemia, our results suggest that those with low HDL or with high ratios of TC/HDL-C or TG/HDL-C belong to a higher risk category for CVD. According these results, the atherogenic index or HDL-C or TG/HDL-C might be included in new CVD risk equations in order to increase the validity of the risk patient assessment and to improve the therapeutic decision-making. However, further research using these risk markers is needed in order to know which one achieves a better risk adjustment model.

In conclusion, this cohort study in a population with high cardiovascular risk shows that HDL-C and TC/HDL-C and TG/HDL-C ratios may be better predictors for mortality and CVD than other lipid parameters commonly used in clinical practice, with relevant practical implications.

## Supporting information

S1 Table Rate ratio and differences (95% CI) for all-cause mortality and CVD hospitalization after a 3.2-year follow-up, comparing the 75^th^ versus 25^th^ percentile of lipid biomarkers concentrations. (DOCX)

S1 Fig Age and sex-adjusted rate ratios for all-cause mortality and CVD hospitalization by serum lipid levels in participants with and without lipid lowering medication. Models are adjusted for age, sex, smoking status (never, former, current), obesity (no, yes), diabetes (no, yes), hypertension (no, yes), chronic kidney disease (no, yes), anti-hypertensive medication (no, yes), glucose-lowering medication (no, yes), lipid-lowering medication (no, yes). Models for specific lipid biomarkers have been additionally adjusted as follows: 1) Total-cholesterol is further adjusted by HDL ≤ 40 for men and ≤ 50 for women (no, yes); 2) HDL-cholesterol is further adjusted by LDL-C ≥130 mg/dL (no, yes); 3) Non-HDL-cholesterol is further adjusted by HDL ≤ 40 for men and ≤ 50 for women (no, yes); 4) LDL-cholesterol is further adjusted by HDL ≤ 40 for men and ≤ 50 for women (no, yes); 5) Non-HDL minus LDL-cholesterol is further adjusted by HDL ≤ 40 for men and ≤ 50 for women (no, yes) and LDL-C ≥ 130 mg/dL (no, yes); 6) Triglycerides is further adjusted by total cholesterol > 200 mg/dL (no, yes) and HDL ≤ 40 for men and ≤ 50 for women (no, yes); 7) Total cholesterol/HDL is further adjusted by total cholesterol (mg/dL); and 7) Triglycerides /HDL is further adjusted by total cholesterol (mg/dL).
(TIF)
