# Correction: Lipid profile, cardiovascular disease and mortality in a Mediterranean high-risk population: The ESCARVAL-RISK study

**DOI:** 10.1371/journal.pone.0205047

**Published:** 2018-09-27

**Authors:** Domingo Orozco-Beltran, Vicente F. Gil-Guillen, Josep Redon, Jose M. Martin-Moreno, Vicente Pallares-Carratala, Jorge Navarro-Perez, Francisco Valls-Roca, Carlos Sanchis-Domenech, Antonio Fernandez-Gimenez, Ana Perez-Navarro, Vicente Bertomeu-Martinez, Vicente Bertomeu-Gonzalez, Alberto Cordero, Manuel Pascual de la Torre, Jose L. Trillo, Concepcion Carratala-Munuera, Salvador Pita-Fernandez, Ruth Uso, Ramon Durazo-Arvizu, Richard Cooper, Gines Sanz, Jose M. Castellano, Juan F. Ascaso, Rafael Carmena, Maria Tellez-Plaza, on behalf of ESCARVAL Study Group

In Table 2, there are incorrect median values in the Q2 column. Please see the corrected Table 2 here.

**Table 2:** Age and sex-adjusted rates of all-cause mortality and CVD hospitalization by quartile of serum lipids.

	Quartile				p-value
	Q1	Q2	Q3	Q4	
**Total cholesterol**					
Median (range), mg/dL	165 (118, 183)	198 (184, 210)	223 (211, 237)	257 (238, 329)	
All-cause mortality					
Cases (person-years)	341 (43, 630.74)	255 (43, 449.59)	184 (41, 295.09)	139 (41, 238.03)	
Rate	60.3	55.5	51.0	49.5	0.027
CHD hospitalization					
Cases (person-years)	609 (42, 385.83)	429 (42, 671.84)	306 (40, 716.51)	322 (40, 637.52)	
Rate	126.0	97.7	80.6	95.2	<0.001
Stroke hospitalization					
Cases (person-years)	504 (42, 683.04)	388 (42, 774.50)	321 (40, 668.79)	297 (40, 714.40)	
Rate	98.3	85.0	84.7	93.9	0.361
**HDL cholesterol**					
Median (range), mg/dL	38 (27, 43)	47.7 (44, 51)	56 (52, 61)	69 (62, 98)	
All-cause mortality					
Cases (person-years)	315 (45, 630.38)	228 (42, 053.36)	191 (42, 115.12)	185 (39, 814.59)	
Rate	66.0	52.9	46.0	50.7	<0.001
CHD hospitalization					
Cases (person-years)	633 (44, 381.46)	435 (41, 240.43)	337 (41, 467.21)	261 (39, 322.61)	
Rate	135.8	103.5	83.7	73.6	<0.001
Stroke hospitalization					
Cases (person-years)	487 (44, 749.58)	378 (41, 346.15)	364 (41, 439.38)	281 (39, 305.62)	
Rate	110.8	90.4	86.3	71.9	<0.001
**Non-HDL cholesterol**					
Median (range), mg/dL	115 (74, 131)	144 (132, 156)	169 (157, 183)	204 (184, 276)	
All-cause mortality					
Cases (person-years)	325 (43, 768.99)	253 (42, 218.93)	192 (42, 588.25)	149 (41, 037.27)	
Rate	58.0	55.9	50.0	52.7	0.154
CHD hospitalization					
Cases (person-years)	558 (42, 614.69)	420 (41, 425.83)	352 (41, 964.77)	336 (40, 406.42)	
Rate	117.6	98.0	88.0	96.4	<0.001
Stroke hospitalization					
Cases (person-years)	477 (42, 867.75)	383 (41, 571.73)	339 (41, 923.08)	311 (40, 478.17)	
Rate	92.1	85.2	85.2	99.8	0.492
**LDL cholesterol**					
Median (range), mg/dL	88 (50, 102)	114 (103, 124)	135.75 (125, 148)	166 (149, 224)	
All-cause mortality					
Cases (person-years)	314 (41, 816.10)	246 (41, 215.15)	203 (43, 304.18)	156 (43, 278.03)	
Rate	60.9	56.0	50.4	49.7	0.015
CHD hospitalization					
Cases (person-years)	577 (40, 679.63)	397 (40, 445.12)	342 (42, 666.73)	350 (42, 620.22)	
Rate	128.4	95.6	83.1	94.4	<0.001
Stroke hospitalization					
Cases (person-years)	460 (40, 965.45)	369 (40, 527.55)	370 (42, 657.15)	311 (42, 690.59)	
Rate	95.5	85.2	90.2	91.1	0.606
**Non-HDL minus LDL cholesterol**					
Median (range), mg/dL	16.6 (7.6, 21.0)	26 (22.0, 30.0)	35 (30.6, 40.3)	48.2 (41.0, 93.0)	
All-cause mortality					
Cases (person-years)	308 (51, 216.52)	239 (42, 451.48)	206 (38, 554.97)	166 (37, 390.47)	
Rate	52.6	52.7	53.8	58.5	0.319
CHD hospitalization					
Cases (person-years)	449 (50, 328.06)	431 (41, 582.03)	391 (37, 851.46)	395 (36, 650.16)	
Rate	85.5	99.8	102.5	118.1	<0.001
Stroke hospitalization					
Cases (person-years)	448 (50, 354.07)	420 (41, 656.99)	335 (37, 963.04)	307 (36, 866.61)	
Rate	81.4	94.8	87.7	101.0	0.013
**Triglycerides**					
Median (range), mg/dL	74 (43, 91)	108 (92, 124)	147 (126, 176)	232 (179, 628)	
All-cause mortality					
Cases (person-years)	256 (43, 230.56)	249 (43, 280.85)	222 (42, 904.17)	192 (40, 197.86)	
Rate	52.8	53.2	52.0	59.2	0.347
CHD hospitalization					
Cases (person-years)	354 (42, 575.48)	400 (42, 462.60)	426 (42, 111.74)	486 (39, 261.89)	
Rate	80.9	91.6	99.6	130.7	<0.001
Stroke hospitalization					
Cases (person-years)	355 (42, 606.01)	400 (42, 519.69)	410 (42, 135.18)	345 (39, 579.85)	
Rate	77.5	88.2	96.0	101.2	<0.001
**Ratio total/HDL cholesterol**					
Median (range), mg/dL	2.96 (2.11, 3.35)	3.70 (3.38, 4.03)	4.42 (4.06, 4.86)	5.58 (4.92, 8.29)	
All-cause mortality					
Cases (person-years)	261 (42, 536.33)	220 (42, 720.99)	238 (42, 608.40)	200 (41, 747.72)	
Rate	53.8	49.1	55.9	58.0	0.276
CHD hospitalization					
Cases (person-years)	370 (41, 798.70)	392 (41, 961.01)	435 (41, 806.98)	469 (40, 845.02)	
Rate	87.2	91.8	102.1	119.8	<0.001
Stroke hospitalization					
Cases (person-years)	386 (41, 761.15)	366 (42, 108.46)	388 (41, 901.78)	370 (41, 069.34)	
Rate	81.3	81.3	92.4	107.3	<0.001
**Ratio TG/HDL cholesterol**					
Median (range), mg/dL	1.20 (0.58, 1.57)	2.00 (1.61, 2.44)	3.05 (2.49, 3.84)	5.49 (3.95, 17.88)	
All-cause mortality					
Cases (person-years)	229 (42, 638.68)	232 (43, 056.65)	241 (42, 876.82)	217 (41, 041.30)	
Rate	51.5	49.4	54.6	61.6	0.041
CHD hospitalization					
Cases (person-years)	286 (42, 105.98)	395 (42, 282.16)	466 (42, 014.74)	519 (40, 008.84)	
Rate	70.3	91.1	107.7	133.2	<0.001
Stroke hospitalization					
Cases (person-years)	321 (42, 083.52)	409 (42, 283.75)	398 (42, 119.24)	382 (40, 354.22)	
Rate	73.2	90.2	92.0	107.3	<0.001

* CVD: cardiovascular disease; CHD: coronary heart disease; HDL: High density lipoprotein; LDL: low density lipoprotein

Age and sex-adjusted rates: events/10,000 person-years
