# Supplementary material for: Lipid profile, cardiovascular disease and mortality in a Mediterranean high-risk population: The ESCARVAL-RISK study
Source: PLoS One. 2017 Oct 18;12(10):e0186196. doi: 10.1371/journal.pone.0186196 (PMC5646809; doi:10.1371/journal.pone.0186196)
Supplement: S1 Table — (DOCX) [file pone.0186196.s001.docx]

**S2 table 1. Rate Ratio and Differences (95% CI) for all-cause mortality and CVD hospitalization after a 3.2-year follow-up, comparing the 75^th^ versus 25^th^ percentile of lipid biomarkers concentrations.**

|  | All-cause mortality | | CHD hospitalization | | Stroke hospitalization | |
| --- | --- | --- | --- | --- | --- | --- |
|  | Model 1 | Model 2 | Model 1 | Model 2 | Model 1 | Model 2 |
| **Total cholesterol ^a^** |  |  |  |  |  |  |
| Rate Ratio | 0.88 (0.78, 1.00) | 0.86 (0.76, 0.98) | 0.84 (0.69, 1.03) | 1.02 (0.82, 1.26) | 0.96 (0.76, 1.22) | 1.08 (0.87, 1.36) |
| Rate Differences  (events/10.000 person-year) | -6.89 (-11.49, -2.29) | -11.53 (-16.48, -6.59) | -17.41 (-24.32, -10.50) | 0.73 (-6.60, 8.06) | -3.90 (-10.49, 2.69) | 6.08 (-0.90, 13.06) |
| **HDL-cholesterol^b^** |  |  |  |  |  |  |
| Rate Ratio | 0.86 (0.76, 0.97) | 0.91 (0.80, 1.03) | 0.71 (0.56, 0.89) | 0.78 (0.62, 0.99) | 0.82 (0.65, 1.03) | 0.87 (0.69, 1.09) |
| Rate Differences  (events/10.000 person-year) | -6.18 (-11.15, -1.21) | -4.05 (-9.31, 1.21) | -29.30 (-35.76, -22.84) | -19.44 (-26.21, -12.68) | -16.17 (-22.56, -9.78) | -10.71 (-17.43, -3.99) |
| **Non-HDL-cholesterol^c^** |  |  |  |  |  |  |
| Rate Ratio | 0.93 (0.82, 1.04) | 0.87 (0.77, 0.98) | 0.93 (0.76, 1.14) | 1.05 (0.85, 1.29) | 1.03 (0.82, 1.30) | 1.10 (0.88, 1.37) |
| Rate Differences  (events/10.000 person-year) | -5.08 (-9.61, -0.56) | -11.15 (-15.91, -6.40) | -8.14 (-15.00, -1.29) | 3.26 (-3.76, 10.28) | 1.45 (-5.12, 8.02) | 6.90 ( 0.12, 13.69) |
| **LDL-cholesterol^d^** |  |  |  |  |  |  |
| Rate Ratio | 0.90 (0.79, 1.01) | 0.86 (0.76, 0.97) | 0.85 (0.70, 1.04) | 1.01 (0.82, 1.25) | 0.97 (0.77, 1.23) | 1.07 (0.86, 1.34) |
| Rate Differences  (events/10.000 person-year) | -6.99 (-11.49, -2.48) | -12.23 (-17.00, -7.46) | -15.80 (-22.52, -9.08) | 0.06 (-6.90, 7.03) | -2.90 (-9.35, 3.55) | 5.23 (-1.48, 11.94) |
| **Non-HDL minus LDL-cholesterol^e^** |  |  |  |  |  |  |
| Rate Ratio | 1.03 (0.92, 1.16) | 0.98 (0.87, 1.10) | 1.19 (0.96, 1.46) | 1.09 (0.88, 1.35) | 1.13 (0.90, 1.43) | 1.06 (0.85, 1.33) |
| Rate Differences  (events/10.000 person-year) | 1.85 (-2.83, 6.52) | -1.28 (-6.14, 3.58) | 14.12 ( 7.70, 20.54) | 6.64 ( 0.05, 13.23) | 8.92 ( 3.05, 14.79) | 4.09 (-2.05, 10.22) |
| **Triglycerides^f^** |  |  |  |  |  |  |
| Rate Ratio | 1.06 (0.94, 1.20) | 1.01 (0.88, 1.16) | 1.26 (1.03, 1.54) | 1.12 (0.89, 1.40) | 1.14 (0.91, 1.44) | 1.00 (0.78, 1.28) |
| Rate Differences  (events/10.000 person-year) | -0.33 (-4.87, 4.21) | -2.94 (-8.01, 2.13) | 20.09 (13.45, 26.74) | 7.89 ( 0.32, 15.46) | 8.33 ( 2.47, 14.18) | -2.69 (-9.37, 3.99) |
| **Total/HDL-cholesterol^g^** |  |  |  |  |  |  |
| Rate Ratio | 1.07 (0.94, 1.21) | 1.08 (0.94, 1.25) | 1.20 (0.96, 1.49) | 1.32 (1.03, 1.69) | 1.17 (0.93, 1.48) | 1.19 (0.92, 1.53) |
| Rate Differences  (events/10.000 person-year) | 1.28 (-3.83, 6.38) | 1.77 (-4.34, 7.88) | 16.07 ( 9.00, 23.14) | 24.36 (16.18, 32.54) | 12.33 ( 5.42, 19.24) | 13.51 ( 5.48, 21.54) |
| **Triglycerides/HDL-cholesterol^h^** |  |  |  |  |  |  |
| Rate Ratio | 1.13 (1.00, 1.28) | 1.08 (0.95, 1.23) | 1.38 (1.12, 1.69) | 1.24 (1.00, 1.55) | 1.21 (0.96, 1.52) | 1.11 (0.88, 1.41) |
| Rate Differences  (events/10.000 person-year) | 2.84 (-1.93, 7.61) | 1.18 (-3.80, 6.17) | 28.78 (22.01, 35.54) | 18.65 (11.60, 25.69) | 13.47 ( 7.34, 19.60) | 6.96 ( 0.33, 13.58) |

* CI: confidence interval; CHD: coronary heart disease; CVD: cardiovascular disease; PAR: population attributable risk; RR: rate ratio; HDL: High density lipoprotein; LDL: low density lipoprotein

Model 1 is adjusted for age and sex. Model 2 is Model 1 further adjusted for smoking status (never, former, current), obesity (no, yes), diabetes (no, yes), hypertension (no, yes), chronic kidney disease (no, yes), anti-hypertensive medication (no, yes), glucose-lowering medication (no, yes), and lipid-lowering medication (no, yes). Models for specific lipid biomarkers have been additionally adjusted as follows: 1) Total-cholesterol**^a^** is further adjusted by HDL ≤ 40 for men and ≤ 50 for women (no, yes); 2) HDL-cholesterol**^b^** is further adjusted by LDL-C ≥ 130 mg/dL (no, yes); 3) Non-HDL-cholesterol**^c^** is further adjusted by HDL ≤ 40 for men and ≤ 50 for women (no, yes); 4) LDL-cholesterol**^d^** is further adjusted by HDL ≤ 40 for men and ≤ 50 for women (no, yes); 5) Non-HDL minus LDL-cholesterol**^e^** is further adjusted by HDL ≤ 40 for men and ≤ 50 for women (no, yes) and LDL-C ≥ 130 mg/dL (no, yes); 6) Triglycerides**^f^** is further adjusted by total cholesterol > 200 mg/dL (no, yes) and HDL ≤ 40 for men and ≤ 50 for women (no, yes); 7) Total cholesterol/HDL**^g^** is further adjusted by total cholesterol (mg/dL); and 7) Triglycerides/HDL**^h^** is further adjusted by total cholesterol (mg/dL)
