# Supplementary figures and images for: Lipid profile, cardiovascular disease and mortality in a Mediterranean high-risk population: The ESCARVAL-RISK study
Source: PLoS One. 2017 Oct 18;12(10):e0186196. doi: 10.1371/journal.pone.0186196 (PMC5646809; doi:10.1371/journal.pone.0186196)

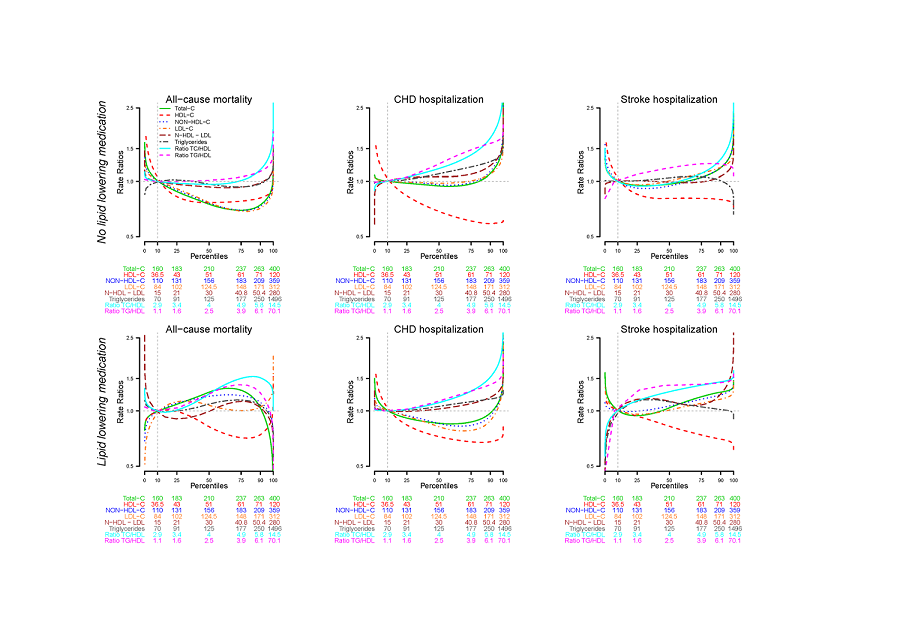

Supplement: S1 Fig — Models are adjusted for age, sex, smoking status (never, former, current), obesity (no, yes), diabetes (no, yes), hypertension (no, yes), chronic kidney disease (no, yes), anti-hypertensive medication (no, yes), glucose-lowering medication (no, yes), lipid-lowering medication (no, yes). Models for specific lipid biomarkers have been additionally adjusted as follows: 1) Total-cholesterol is further adjusted by HDL ≤ 40 for men and ≤ 50 for women (no, yes); 2) HDL-cholesterol is further adjusted by LDL-C ≥130 mg/dL (no, yes); 3) Non-HDL-cholesterol is further adjusted by HDL ≤ 40 for men and ≤ 50 for women (no, yes); 4) LDL-cholesterol is further adjusted by HDL ≤ 40 for men and ≤ 50 for women (no, yes); 5) Non-HDL minus LDL-cholesterol is further adjusted by HDL ≤ 40 for men and ≤ 50 for women (no, yes) and LDL-C ≥ 130 mg/dL (no, yes); 6) Triglycerides is further adjusted by total cholesterol > 200 mg/dL (no, yes) and HDL ≤ 40 for men and ≤ 50 for women (no, yes); 7) Total cholesterol/HDL is further adjusted by total cholesterol (mg/dL); and 7) Triglycerides /HDL is further adjusted by total cholesterol (mg/dL). (TIF) [file pone.0186196.s002.tif]
